# Development and evaluation of a concise food list for use in a web-based 24-h dietary recall tool

**DOI:** 10.1017/jns.2017.49

**Published:** 2017-08-29

**Authors:** Katie Evans, Áine Hennessy, Janette Walton, Claire Timon, Eileen Gibney, Albert Flynn

**Affiliations:** 1 School of Food and Nutritional Sciences, University College Cork, Cork, Republic of Ireland; 2 UCD Institute of Food and Health, University College Dublin, Belfield, Dublin, Republic of Ireland

**Keywords:** 24-h Recall, Concise food lists, Dietary assessment, Foodbook24, Food composition data, EAR, estimated average requirement, NANS, Irish National Adult Nutrition Survey, UL, upper intake level

## Abstract

Foodbook24 is a self-administered web-based 24-h dietary recall tool developed to assess food and nutrient intakes of Irish adults. This paper describes the first step undertaken in developing Foodbook24 which was to limit participant burden by establishing a concise list of food items for inclusion in the tool. The aim of the present study was to evaluate whether using a concise food list (as opposed to an extensive list) with generic composition data would influence the estimates of nutrient intakes in a nationally representative sample of Irish adults. A 2319-item food list generated from the Irish National Adult Nutrition Survey (NANS) (2008–2010) (*n* 1500) was used as the basis for a shortened food list for integration into the tool. Foods similar in nutritional composition were recoded with a generic type food code to produce a concise list of 751 food codes. The concise food list was applied to the NANS food consumption dataset and intake estimates of thirty-five nutrients were compared with estimates derived using the original extensive list. Small differences in nutrient intakes (<6 %) with limited effect size (Cohen's *d* < 0·1) were observed between estimates from both food lists. The concise food list showed strong positive correlations (*r*_s_ 0·9–1·0, *n* 1500, *P* < 0·001) and a high level of agreement with the extensive list (80–97 % of nutrient intakes classified into the same tertile; >90% of intakes similarly categorised according to dietary reference values). This indicates that a concise food list is suitable for use in a web-based 24-h dietary recall tool for Irish adults.

Valid estimation of habitual nutrient intakes of a population group relies on dietary assessment methods which collect detailed food intake data that are linked to precise food composition data^(^^1^^,^^2^^)^. As there is no ‘gold standard’ method of dietary intake assessment, the method chosen largely depends on the research question, population group, available resources, and the foods and nutrients of interest^(^^2^^,^^3^^)^. In order to characterise the habitual dietary exposures of an adult population group, multiple non-consecutive 24-h dietary recalls may represent a useful tool as they are capable of providing relatively detailed dietary intake data with minimal bias^(^^4^^–^^9^^)^. The immediacy of the recall period usually enables the participant to successfully recall most of their daily intake and the retrospective nature of the data collection reduces the risk of the participant altering their dietary behaviour. Furthermore, 24-h recalls place relatively little burden on the participant in comparison with prospective methods^(^^2^^)^.

Development of web-based, self-administered 24-h dietary recalls may provide the opportunity for efficient and cost-effective dietary assessment^(^^4^^–^^6^^,^^8^^–^^10^^)^. As with any other dietary assessment method, self-administered web-based tools are subject to many of the biases intrinsic to interviewer-administered methods. The self-reporting nature of 24-h recalls can result in misreporting of food and energy intake and participant fatigue can also be an issue^(^^2^^)^. Due to the absence of an interviewer, the process and user interface must be simple and user-friendly. Behind the user interface, the foods selected by the participant must be fully auto-coded and linked to a comprehensive food composition database, which in turn calculates nutrient intake from the food item^(^^6^^,^^11^^–^^13^^)^.

Foodbook24 is a self-administered web-based 24-h dietary recall tool developed to assess food and nutrient intakes in a population of Irish adults. The purpose and scope of this tool are to allow researchers to simply and quickly estimate the daily intake of foods, and macro- and micronutrients in Irish adult populations. It is therefore important to limit participant burden as far as possible by limiting the number of food items available for the participant to choose. A more extensive food list would be necessary to conduct exposure assessment of bioactive and non-nutritional compounds, food chemicals and food additives or conduct risk–benefit analysis of fortification strategies. This paper describes the first step undertaken in developing Foodbook24 which was to establish a concise list of food items for inclusion in the tool. The aim of the present study was to evaluate whether using a concise list of foods (as opposed to an extensive list), together with the associated generic food composition data, would influence the estimates of nutrient intake in Irish adults, using data from a nationally representative nutrition survey of adults in Ireland as a case study. Further studies examining the validity and acceptability of Foodbook24 are currently in progress.

## Methods

### Study design and study population

The National Adult Nutrition Survey (NANS)^(^^14^^)^ food consumption dataset was used as the source of food items and composition, to be integrated into the Foodbook24 tool. The NANS, a nationally representative, cross-sectional nutrition survey, was conducted by the Irish Universities Nutrition Alliance between 2008 and 2010 with the aim of establishing a dataset of habitual food and beverage consumption of adults aged 18 to 90 years living in Ireland. Detailed health and lifestyle, eating behaviour and anthropometric data were also collected.

### Dietary assessment methodology in the National Adult Nutrition Survey

A 4-d semi-weighed food record was completed by 1500 adults and the dietary intake data recorded by participants were converted to nutrient intakes using the Weighed Intake Software Package (WISP; Tinuviel Software), which encompasses UK food composition tables^(^^15^^–^^24^^)^ and The Irish Food Composition Database (IFCDB)^(^^25^^)^. The IFCDB has been consistently updated during each Irish national nutrition survey to reflect the most recent composition data for fortified foods, nutritional supplements, composite dishes and Irish brands consumed which were not adequately characterised by UK food composition tables. The accuracy of food composition, as well as consumption, was aided by asking participants to retain food packaging during the survey period. It is worth noting that if a NANS participant reported consuming a homemade composite dish, the recipe was obtained from the participant and a new food code and accompanying food composition value were generated, if the dish was not adequately characterised by existing food codes. At its most disaggregated level, the NANS dataset consists of 133 068 rows of food intake data, comprised of 7653 branded food items which were described by 2552 food codes, of which 233 were nutritional supplements.

### Derivation of the concise food list for Foodbook24

The Foodbook24 user interface aims to provide the participant with a finite number of food item options without requiring brand information, that are linked to food composition data, allowing the automatic calculation of nutrient intakes and thus reducing the time required for data entry and data analysis by the researcher. With the overall aim of reducing participant burden, the objective of the present study was to develop a concise list of food codes available for selection by the Foodbook24 user and evaluate the impact of using this concise list on the estimates of nutrient intakes in the Irish population.

The 2319 food codes used in the NANS were initially evaluated at a food-group level and the food codes that described similar food items/food types were combined and the nutritional composition of the most frequently used food code within a food type was selected for inclusion in the Foodbook24 tool. An excerpt of these recoding exercises is available in Supplementary Table S1. Ten different brands of plain/flavoured full-fat yoghurt were reported during the 4-d recording period by participants of the NANS. The frequency of consumption was examined and ranged from one to 396 eating occasions during the survey period. The nutritional composition of the most commonly consumed yoghurt (in this case the yoghurt consumed 396 times) was brought forward for inclusion in the concise food list (Supplementary Table S1).

A small number of food items (*n* 113) were excluded from this recoding exercise and the original food code and associated nutritional composition were retained. Of these 113 food items, forty-eight items were recorded as an ingredient of a recipe or composite dish, e.g. cornflour; sixty-two items were unusual food items that were consumed infrequently (fifty-seven of which were consumed less than ten times by the total sample); three food codes were unique to a study participant or were not similar enough to be recoded or aggregated with other food items.

### Statistical analysis

To test the suitability of this concise food list for use in the Foodbook24 tool, we evaluated the level of agreement between estimates of nutrient intake using the concise food list (*n* 751 food codes) together with generic food composition data with the extensive complete food list used in the NANS (*n* 2319 food codes), when applied to the NANS food consumption dataset. The distributions of nutrient intakes were assessed for normality using Kolmogorov–Smirnov tests and the natural log and square root transformations were applied, where appropriate. All statistical analyses were performed in SPSS^©^ version 21 (IBM). An *α* of 0·01 was selected to compensate for a possible increased risk of type 1 errors due to the use of multiple tests.

We evaluated the level of agreement between estimates of intake derived from the concise food list and extensive food list composition sources in a number of ways.

#### Association between estimates

Estimates of nutrient intake from food sources only were calculated using both the extensive and concise list of food codes and a paired-samples *t* test, or a Wilcoxon signed-rank test where appropriate, were performed to identify significant differences in estimates of intake. Cohen's *d*^(^^26^^)^ was used to examine the effect size of the differences between both the extensive food list and the concise food list estimates. The percentage difference between estimates of intake from food sources was calculated by subtracting the mean intake estimate using the old (extensive list) from the new (concise list) mean estimate of intake and dividing this value by the mean old (extensive list) estimate and multiplying by 100.

#### Cross-classification analysis

Cross-classification analysis quantified the level of agreement between categorisation of estimates (from food sources only) into thirds (tertiles) of the intake distribution. The percentage of participants categorised into the same tertile of intake was calculated and the level of agreement quantified using Cohen's *κ* statistic. For macronutrients, the percentages of participants meeting the recommendations for protein^(^^27^^)^, carbohydrate^(^^28^^)^, total fat^(^^29^^)^, saturated fat^(^^30^^)^ and dietary fibre^(^^28^^)^ proposed by the European Food Safety Authority (EFSA) and the UK Department of Health were identified. Using the cut-point method proposed by Carriquiry^(^^31^^)^ and described by the US Institute of Medicine^(^^32^^)^, the proportion of NANS participants with micronutrient intakes (from all sources) below the estimated average requirement (EAR)^(^^30^^,^^33^^,^^34^^)^ and above the tolerable upper intake level (UL)^(^^35^^–^^37^^)^ were calculated and the level of agreement was analysed using Cohen's *κ* statistic. Given the large impact of nutritional supplements on micronutrient intakes^(^^38^^)^ and on estimates of intake relative to dietary reference values^(^^39^^)^, Foodbook24 will approach supplement use in an open-ended manner, allowing the user to enter the brand of nutritional supplement they use into the tool. Thus, for the purpose of this study, we did not reduce the number of nutritional supplement food codes. As misreporting of food and energy intake and particularly under-reporting is a known issue in all methods of dietary assessment, under-reporters of energy intake (29·3 %), identified as having an energy intake:BMR^(^^40^^)^ ratio of <1·1^(^^41^^)^, were excluded from the EAR analysis.

## Results

Using the criteria described above, the number of food codes selected for use in the concise food list was reduced from 2319 to 751 food codes, representing a reduction of 68 % (Table 1). The largest reductions in the number of food items were made for ‘butter, spreading fats and oils’, ‘eggs and egg dishes’ and ‘bread and rolls’ (78, 77 and 75 %, respectively). The number of food items in all other food groups was reduced by 53–73 %.

**Table 1.** List of all food codes included in the National Adult Nutrition Survey and shortened list of food codes developed for use in the Foodbook24 tool

Food group	Extensive food list (*n* 2319)	Concise food list (*n* 751)
Grains, rice, pasta and pizza	110	42
Bread and rolls	75	19
Breakfast cereals	77	33
Biscuits, cakes and pastries	133	38
Milk and yoghurt	70	19
Creams, ice-creams and chilled desserts	92	36
Cheeses	45	17
Butter, spreading fats and oils	36	8
Eggs and egg dishes	31	7
Potatoes and potato products	60	18
Vegetable and vegetable dishes	271	103
Fruit and fruit juices	179	46
Fish and fish dishes	160	43
Meat and meat products	510	139
Beverages	114	48
Sugars, confectionery, preserves and savoury snacks	120	57
Soups, sauces and miscellaneous foods	180	56
Nuts, seeds, herbs and spices	56	22

Small differences in estimates of mean nutrient intakes from ‘food sources only’ were observed when the concise food list was applied to the NANS food consumption database (Table 2), where the magnitude of the difference in energy, protein, carbohydrate, starch, sugar, non-milk sugars, total fat, polyunsaturated fat and alcohol intakes was <1 % at a population level. The largest differences were observed for saturated fat (+2·0 %) and monounsaturated fat (−2·3 %). The percentage difference between estimates of dietary fibre was −1·4 %. Differences in estimates of micronutrient intakes were <2 % for retinol, carotene, vitamin A, niacin, folate, folic acid, biotin, pantothenate, vitamin C, Ca, Mg, P, Zn and Cu. Larger differences between estimates were observed for vitamin D (−3·4 %), vitamin E (−5·0 %), vitamin B_1_ (−5·8 %), vitamin B_2_ (−3·4 %), Fe (−2·6 %), Na (−2·5 %) and K (−2·1 %). The practical significance of the difference between estimates of intake, as defined by Cohen's convention for effect size, was small for all nutrients, where all values were <0·1.

**Table 2.** Differences in the mean daily intake of energy and nutrients from food sources only in Irish adults aged 18–90 years (*n* 1500) from the National Adult Nutrition Survey using both an extensive (*n* 2319) and a concise (*n* 751) food list (Mean values and standard deviations, and percentage differences)

Nutrient	Extensive food list (*n* 2319)		Concise food list (*n* 751)				
	Mean	sd	Mean	sd	% Difference (%)*	*P*	Cohen's *d*^(^^26^^)^
Energy							
kcal	2008	656	2008	653	0·0	0·225‡	<0·001
kJ	8401	2745	8401	2732	0·0	0·225‡	<0·001
Protein (g)	83·3	26·9	83·1	26·6	−0·3	0·106‡	0·010
Carbohydrate (g)	228	79	228	78	−0·2	0·001‡	0·006
Total sugars (g)	90·3	43·1	90·0	42·6	−0·3	0·223†	0·006
Non-milk sugars (g)	76·6	39·7	76·3	39·2	−0·4	0·178†	0·008
Starch (g)	134	48	134	48	0·1	0·581†	0·002
Total fat (g)	75·7	29·4	75·5	29·1	−0·3	0·224†	0·007
Saturated fat (g)	29·7	13·0	30·3	13·0	2·0	<0·001‡	0·046
Monounsaturated fat (g)	27·7	11·4	27·0	11·0	−2·3	<0·001‡	0·057
Polyunsaturated fat (g)	13·3	6·5	13·3	6·4	0·3	0·474‡	0·005
Dietary fibre (g)	19·1	7·9	18·9	7·7	−1·4	<0·001‡	0·035
Alcohol (g)	15·7	24·4	15·7	24·3	−0·2	<0·001‡	0·001
Retinol (μg)	410	623	405	633	−1·3	<0·001‡	0·008
Carotene (μg)	3680	3177	3651	2945	−0·8	<0·001‡	0·009
Vitamin A (μg)	1024	831	1014	821	−1·0	0·623‡	0·012
Vitamin D (μg)	3·2	2·6	3·1	2·5	−3·4	0·015†	0·043
Vitamin E (mg)	9·4	4·9	8·9	4·6	−5·0	<0·001†	0·098
Vitamin B_1_ (mg)	1·8	2·2	1·9	4·8	5·8	<0·001‡	0·027
Vitamin B_2_ (mg)	1·9	0·8	1·8	0·8	−3·4	<0·001†	0·078
Niacin (mg)	24·2	10·7	24·1	10·7	−0·2	0·674†	0·004
Vitamin B_6_ (mg)	2·7	1·4	2·7	1·4	1·4	0·006†	0·026
Vitamin B_12_ (μg)	5·5	3·8	5·6	3·9	1·9	0·001†	0·026
Folate (μg)	317	150	314	146	−0·8	0·067†	0·018
Folic acid (μg)	79	102	78	97	−1·1	<0·001‡	0·009
Biotin (μg)	37·4	19·5	37·1	21·3	−0·7	0·006†	0·012
Pantothenate (mg)	5·9	2·4	5·9	2·4	0·6	0·031†	0·014
Vitamin C (mg)	79	52	78	54	−2·0	<0·001‡	0·030
Ca (mg)	899	372	897	370	−0·2	0·562‡	0·006
Mg (mg)	282	102	277	99	−1·9	<0·001‡	0·053
P (mg)	1378	461	1365	453	−1·0	<0·001†	0·029
Zn (mg)	9·2	3·5	9·1	3·4	−0·9	<0·001‡	0·023
Cu (mg)	1·1	0·6	1·0	0·6	−1·3	0·002‡	0·025
Fe (mg)	12·0	5·1	11·7	5·0	−2·6	<0·001‡	0·062
K (mg)	3041	969	2979	932	−2·1	<0·001†	0·066
Na (mg)	2497	903	2559	937	2·5	<0·001†	0·068

* Calculated as the difference of the mean intake (new code – old code) divided by the mean intake using the old food codes and multiplied by 100.

† Paired-samples *t* test on transformed normally distributed variables.

‡ Wilcoxon signed-rank test on non-normally distributed variables.

When mean estimates of nutrient intake were calculated from all sources (including supplements) (Supplementary Table S2), the differences in mean daily intake using the extensive and concise food lists were even smaller (<4 %) than those observed from food sources only. However, as with the estimates from food sources only, the practical significance of the difference between estimates of intake from all sources (including supplements) was small.

The association between estimates of nutrient intake (from food sources only) was assessed by calculating the percentage of participants who were categorised into the same third of the intake distribution based on the extensive (*n* 2319) and concise (*n* 751) food lists (Table 3). The majority of participants were classified into the same third of the nutrient intake distribution when using the concise and extensive food lists, ranging from 80 % for vitamin E to 97 % for alcohol.

**Table 3.** Association between estimates of nutrient intake from food sources only using the extensive (*n* 2319) and concise (*n* 751) food lists

Nutrient	Proportion in the same tertile (%)	Cohen's *κ*	*P*
Energy (kJ or kcal)	95·6	0·934	<0·001
Protein (g)	92·8	0·892	<0·001
Carbohydrate (g)	94·5	0·918	<0·001
Total sugars (g)	92·8	0·892	<0·001
Non-milk sugars (g)	90·7	0·861	<0·001
Starch (g)	92·7	0·890	<0·001
Total fat (g)	87·9	0·818	<0·001
Saturated fat (g)	88·4	0·826	<0·001
Monounsaturated fat (g)	85·3	0·779	<0·001
Polyunsaturated fat (g)	82·2	0·733	<0·001
Dietary fibre (g)	90·7	0·860	<0·001
Alcohol (g)	96·8	0·951	<0·001
Retinol (μg)	87·2	0·808	<0·001
Carotene (μg)	84·9	0·774	<0·001
Vitamin A (μg)	84·4	0·766	<0·001
Vitamin D (μg)	85·0	0·775	<0·001
Vitamin E (mg)	79·7	0·695	<0·001
Vitamin B_1_ (mg)	84·3	0·764	<0·001
Vitamin B_2_ (mg)	91·4	0·871	<0·001
Niacin (mg)	89·9	0·849	<0·001
Vitamin B_6_ (mg)	86·8	0·802	<0·001
Vitamin B_12_ (μg)	86·0	0·790	<0·001
Folate (μg)	86·0	0·790	<0·001
Folic acid (μg)	83·9	0·759	<0·001
Biotin (μg)	86·6	0·799	<0·001
Pantothenate (mg)	88·2	0·823	<0·001
Vitamin C (mg)	85·6	0·784	<0·001
Ca (mg)	92·4	0·886	<0·001
Mg (mg)	91·4	0·871	<0·001
P (mg)	92·9	0·894	<0·001
Fe (mg)	89·9	0·849	<0·001
Zn (mg)	89·2	0·838	<0·001
Cu (mg)	84·1	0·761	<0·001
K (mg)	90·8	0·862	<0·001
Na (mg)	86·3	0·795	<0·001

The proportion of adults with adequate protein intakes was 93 and 92 % using both the concise and the extensive food lists, respectively, and 98 % of participants were classified similarly by the two methods (*κ* 0·883; *P* < 0·001). Using both lists, the proportions of participants classified as meeting dietary reference values for macronutrients were similar (carbohydrate, 52 % meeting dietary reference value where 91 % of participants were categorised similarly (*κ* 0·813; *P* < 0·001); and for total fat, 59 % meeting dietary reference value, where 90 % were similarly classified (*κ* 0·787; *P* < 0·001)). Though a lower proportion of participants met the UK Department of Health recommendation for saturated fat using the concise food list (15 %) than the extensive food list (18 %), the level of agreement between estimates was still relatively good (92 % similarly classified; *κ* 0·720; *P* < 0·001). The proportions of participants with intakes of dietary fibre below the adequate intake of 25 g/d were similar using both lists (81 and 82 %, respectively; 96 % of participants similarly classified; *κ* 0·875; *P* < 0·001) (Table 4).

**Table 4.** Percentage of participants meeting dietary reference values for macronutrients^(^^27^^–^^30^^)^ and dietary fibre^(^^28^^)^ intake from all sources including nutritional supplements

	% Meeting recommendations using extensive food list	% Meeting recommendations using concise food list	% Classified into the same category	Cohen's *κ* statistic
Protein (≥0·66 g/kg body weight)	92·7	92·2	98·4	0·883
Carbohydrate (%E ≥ 45 and ≤60)	52·3	52·3	90·7	0·813
Total fat (%E ≥ 20 and ≤35)	58·5	59·3	89·7	0·787
Saturated fat (%E ≤ 10)	17·7	14·6	92·4	0·720
Fibre intake ≥25 g/d	19·1	18·1	96·2	0·875

%E, percentage total energy intake.

There was a high level of agreement between the proportion of individuals identified with intakes below the EAR and above the UL using both the concise and extensive food lists, where 96–100 % of participants were classified into the same category of nutrient adequacy (*κ* 0·755–1·000) (Table 5). With respect to the proportion of individuals with intakes above the UL, there were minimal differences between the estimates derived from the extensive or concise food lists (Table 6). In terms of classification of individuals into categories of excessive intake, >99·7 % of individuals were classified into the same category of excessive intake using the concise food list.

**Table 5.** Classification of participants identified with intakes below the estimated average requirement (EAR)^(^^30^^,^^33^^,^^34^^)^ (excluding under-reporters^(^^40^^,^^41^^)^) from all sources including nutritional supplements

Nutrient	% <EAR using extensive food list	% <EAR using concise food list	% Classified into same category	*κ*
Vitamin A	12·2	11·5	95·8	0·797
Vitamin D	90·0	90·8	97·7	0·870
Thiamin	0·0	0·0	100·0	1·000
Riboflavin	2·1	2·5	99·6	0·915
Niacin	1·3	1·3	99·6	0·855
Vitamin B_6_	0·4	0·4	100·0	1·000
Vitamin B_12_	1·2	0·8	99·2	0·567
Folate	3·8	3·4	98·5	0·782
Vitamin C	6·2	7·0	97·0	0·755
Ca	4·1	4·1	98·7	0·876
Mg	7·7	7·6	97·5	0·903
Zn	8·3	8·3	97·4	0·827
Fe	18·0	18·0	97·3	0·909

**Table 6.** Classification of participants identified with intakes above the tolerable upper intake level (UL)^(^^35^^-^^37^^)^ from all sources including nutritional supplements

Nutrient	% >UL using extensive food list	% >UL using concise food list	% Classified into same category	*κ*
Retinol	0·9	0·9	100·0	1·000
Vitamin D	0·1	0·1	100·0	1·000
Vitamin E	0·6	0·6	100·0	1·000
Pre-formed niacin	0·0	0·0	100·0	1·000
Vitamin B_6_	1·7	1·7	100·0	1·000
Folic acid	0·5	0·5	99·9	0·933
Vitamin C	0·2	0·2	100·0	1·000
Ca	0·3	0·3	99·9	0·749
Fe	1·8	1·8	100·0	1·000
Zn	1·5	1·7	99·7	0·915
P	0·0	0·0	100·0	1·000
Cu	1·6	1·6	99·9	0·958

## Discussion

The aim of the present study was to describe one of the initial stages in the development of the Foodbook24 tool: the establishment of a concise food list and evaluation of the impact of using this concise food list on estimates of energy and nutrient intake in a nationally representative reference sample of Irish adults. This is an essential first component in the development of the Foodbook24 tool given the critical role that food composition datasets play in obtaining a reliable estimate of nutrient intake^(^^42^^)^. The results of these analyses indicate that the recalculation of energy and nutrient intake using the concise food list resulted in small differences in summary estimates of intake for energy, macronutrients and micronutrients in this reference sample of adults. However, some notable exceptions exist, which will be discussed below.

The present study has demonstrated that even with a considerable reduction in the number of food codes (68 %), the differences between population estimates of intake derived by the concise food list in comparison with the extensive food list were small (less than 6 %). While the differences in estimates of intake were statistically significant for macronutrients such as saturated and monounsaturated fats, dietary fibre, and for a number of micronutrients also, the practical significance of these differences, as defined by Cohen's convention for effect size, was not practically meaningful.

There was a strong positive association between the estimates of daily nutrient intake from food sources and all sources derived using the two food lists. We observed a high proportion of participants classified into the same category of the nutrient intake distribution using the concise food list, which indicated a high level of agreement between the estimates derived from both lists. For energy, protein, carbohydrate, sugars and dietary fibre, over 90 % of participants were classified into the same third of intake. However, we observed a lower degree of cross-classification for estimates of total fat, saturated, monounsaturated and polyunsaturated fats (82–88 % of participants were classified into the same tertile).

It is important to note that in the NANS a systematic approach to assign accurate and reliable fatty acid composition data from multiple sources, i.e. published food tables, papers, packaging information and industry data^(^^43^^)^, was utilised. The observed differences in absolute intakes of saturated and monounsaturated fat in the present study are attributable to loss of subtle fatty acid composition detail as a result of using a generic approach to fatty acid composition data. Though fatty acid intake data show the greatest level of discrepancy, the ability of the concise list to characterise saturated intakes relative to dietary reference values is still relatively good at a population level (saturated fat ≤10 % total energy: 18 *v.* 15 %, *κ* 0·72).

Similar to differences in absolute intakes, we found that the lower degree of cross-classification observed for fats was attributable to changes in fatty acid composition data, particularly for meat and meat products (saturated and polyunsaturated fat intake), and butters, spreading fats and oils (monounsaturated and polyunsaturated fat intake). The recoding of food items such as soups and stir-in sauces had a modest impact on estimates of total fat, saturated and monounsaturated fat intake. Should the Foodbook24 tool be used for epidemiological research with a particular focus on dietary fat intake, we would advise that the above food groups be disaggregated to some extent to capture the subtle but important differences in the monounsaturated and polyunsaturated fat content of certain food groups.

Similarly, estimates of dietary fibre intakes in the NANS were obtained using a systematic approach to food composition data, ensuring that the most accurate and reliable information from published sources and the food industry are incorporated into dietary survey databases^(^^44^^)^. While a small difference between estimates was observed (−1·4 %), the practical significance of the difference was minimal, and the level of cross-classification was quite good for absolute intakes (91 % classified into the same third of the intake distribution; *κ* 0·86) and for the proportion of adults meeting the EFSA reference value for dietary fibre (19 and 18 % of adults with intake ≥25 g/d).

An important function of collecting dietary intake data in large samples of the population is to evaluate the prevalence of nutrient adequacy and risk for excessive intakes. With respect to absolute intakes of micronutrients, we found that 80–91 and 84–93 % of participants were classified into the same third of vitamin and mineral intake distributions (from food sources), respectively. Application of the concise food list to the NANS database did not significantly alter the proportion of the population with intakes below the EAR or above the UL, with over 96 and 99 % of individuals classified into the same categories of nutrient adequacy and risk of excessive intake, respectively. As the concise short list does not include brand-level information we advise interpreting data on micronutrient adequacy and potential for excessive intake with some level of caution.

While it is well recognised that the quality and completeness of food composition data have a major influence on the reliability of estimates of nutrient intake, few studies, including dietary assessment method development studies, have presented data on the development of food lists or evaluation of their performance. Extensive food lists and associated food composition databases (often including brand information) are incorporated into a number of existing online dietary assessment tools^(^^4^^,^^6^^,^^8^^,^^13^^,^^45^^)^. However, in the present study a shortened, more concise, food list was developed to limit participant burden by reducing the number of foods that the participant has to choose from. To the best of our knowledge, there are a limited number of studies that have evaluated the impact of reducing the number of food codes and using generic food composition data. Freese *et al.*^(^^46^^)^ recently developed a short food list (*n* 246) for use in a 24-h recall tool which forms part of a blended strategy to estimate usual nutrient intakes. Freese *et al.*^(^^46^^)^ identified food items consumed in the German national nutrition surveys, NVS I and NVS II, which accounted for 75 % of the variation of intakes of twenty-seven nutrients using a stepwise linear regression approach, supplemented with additional checks for completeness.

The results of the present study show that a concise food list developed for use in the Foodbook24 tool shows good performance in assessing population food and nutrient intakes in Irish adults. However, there are some limitations on the ability of the concise list to estimate intakes of fatty acids and some micronutrients at an individual level. It is worth noting again that the intention of the shortened food list was to limit participant burden by reducing the number of foods the participant has to choose from. The concise food list developed has a number of key strengths. The food list items were derived from nationally representative food consumption data and are therefore reflective of foods that are actually consumed by the Irish adult population. The NANS food consumption dataset is very detailed, where each food item has been recorded and described to brand level. In the development of the concise food list, the number of food and beverage items was considerably reduced. As with all food composition data, it would be important that the concise food list is monitored and updated to reflect any changes in food consumption patterns and to incorporate new foods on the market.

### Conclusion

Our evaluation of the concise food list developed and applied to the NANS food consumption dataset as a case study has shown a high level of agreement with a more extensive list with respect to estimating nutrient intakes and categorising nutrient intakes according to dietary reference values. This indicates that a concise food list is suitable for estimating food and nutrient intakes of Irish adults and can be incorporated into a web-based 24-h dietary recall tool, provided the purpose of the analysis fits within the recognised scope and coverage of the concise list.

## Supplementary material

For supplementary material accompanying this paper visit http://dx.doi.org/10.1017/jns.2017.49. click here to view supplementary material
