# Supplementary material for: Development and evaluation of a concise food list for use in a web-based 24-h dietary recall tool
Source: J Nutr Sci. 2017 Aug 29;6:e46. doi: 10.1017/jns.2017.49 (PMC5672317; doi:10.1017/jns.2017.49)
Supplement: Supplementary file 1 [file S2048679017000490sup001.doc]

| **Supplementary Table S1.** Example of recoding exercise to select one generic food code for whole milk yoghurts | | | |
| --- | --- | --- | --- |
| Original food code | Description of food | Frequency of consumption | Food code to be used for concise food list |
| 5501 | Yoghurt, luxury, containing chocolate | 9 | 12375 |
| 5502 | Yoghurt, luxury, containing caramel | 1 |
| 5503 | Yoghurt, containing hazelnuts | 25 |
| 5515 | Yoghurt, custard-style | 7 |
| 5537 | Yoghurt, containing cereal/granola | 23 |
| 5551 | Yoghurt, containing strawberry | 29 |
| 12184 | Whole milk yoghurt (plain) | 88 |
| 12186 | Whole milk yoghurt (organic) | 33 |
| 12187 | Whole milk goats yoghurt | 9 |
| 12375 | Whole milk fruit with yoghurt | 396 |

| **Supplementary Table S2.** Comparing the differences in the mean daily intake (MDI) of total energy and nutrients from all sources in Irish adults aged 18-90yrs (n=1500) from the National Adult Nutrition Survey (NANS) using both an extensive (n=2319) and a concise (n=751) food list | | | | | | | |
| --- | --- | --- | --- | --- | --- | --- | --- |
| Nutrient | Extensive food list (n =2,319) | | Concise food list (n=751) | | % difference* | *P* | Cohen's *d*^(26)^ |
|
|  | **Mean** | **SD** | **Mean** | **SD** | **%** |  |  |
| Energy (kcal) | **2014** | 657 | **2014** | 653 | 0.0 | 0.987 | <0.001 |
| Protein (g) | **83.9** | 27.9 | **83.7** | 27.6 | -0.3 | 0.027 | 0.009 |
| Carbohydrate (g) | **229** | 79 | **228** | 78 | -0.2 | 0.025 | 0.006 |
| Total sugars (g) | **90.4** | 43.2 | **90.2** | 42.6 | -0.3 | 0.086 | 0.006 |
| Non-milk sugars (g) | **76.7** | 39.7 | **76.4** | 39.2 | -0.4 | 0.063 | 0.008 |
| Starch (g) | **134** | 48 | **134** | 48 | 0.1 | 0.581 | 0.002 |
| Total fat (g) | **75.9** | 29.3 | **75.7** | 29.1 | -0.3 | 0.199 | 0.007 |
| Saturated fat (g) | **29.8** | 12.9 | **30.4** | 13.0 | 2.0 | <0.001 | 0.047 |
| Monounsaturated fat (g) | **27.7** | 11.4 | **27.1** | 11.0 | -2.3 | <0.001 | 0.057 |
| Polyunsaturated fat (g) | **13.4** | 6.5 | **13.5** | 6.5 | 0.3 | 0.631 | 0.005 |
| Dietary fibre (g) | **19.2** | 8.0 | **18.9** | 7.8 | -1.4 | <0.001 | 0.034 |
| Alcohol (g) | **15.7** | 24.4 | **15.7** | 24.3 | -0.2 | 0.334 | 0.001 |
| Retinol (µg) | **502** | 718 | **497** | 727 | -1.0 | 0.081 | 0.007 |
| Carotene (µg) | **3750** | 3233 | **3721** | 3009 | -0.8 | 0.368 | 0.009 |
| Vitamin A (µg) | **1127** | 924 | **1117** | 912 | -0.9 | 0.087 | 0.011 |
| Vitamin D (µg) | **4.6** | 6.0 | **4.5** | 6.0 | -2.4 | <0.001 | 0.018 |
| Vitamin E (mg) | **15.2** | 44.8 | **14.8** | 44.8 | -3.0 | <0.001 | 0.010 |
| Vitamin B1 (mg) | **3.0** | 7.9 | **3.1** | 9.2 | 3.4 | 0.154 | 0.012 |
| Vitamin B2 (mg) | **3.1** | 7.2 | **3.0** | 7.2 | -2.1 | <0.001 | 0.009 |
| Niacin (mg) | **28.0** | 19.8 | **28.0** | 19.8 | -0.1 | 0.456 | 0.002 |
| Vitamin B6 (mg) | **4.0** | 7.3 | **4.0** | 7.3 | 0.9 | 0.001 | 0.005 |
| Vitamin B12 (µg) | **7.5** | 30.0 | **7.6** | 30.0 | 1.4 | 0.001 | 0.003 |
| Folate (µg) | **370** | 326 | **368** | 323 | -0.7 | 0.066 | 0.008 |
| Folic acid (µg) | **132** | 309 | **131** | 307 | -0.7 | 0.499 | 0.003 |
| Biotin (µg) | **49.5** | 75.6 | **49.2** | 76.0 | -0.5 | 0.344 | 0.003 |
| Pantothenate (mg) | **7.9** | 11.6 | **7.9** | 11.6 | 0.4 | 0.070 | 0.003 |
| Vitamin C (mg) | **124** | 225 | **123** | 225 | -1.3 | <0.001 | 0.007 |
| Calcium (mg) | **944** | 416 | **942** | 414 | -0.2 | 0.203 | 0.005 |
| Magnesium (mg) | **291** | 110 | **286** | 107 | -1.8 | <0.001 | 0.049 |
| Phosphorous (mg) | **1382** | 463 | **1369** | 454 | -1.0 | <0.001 | 0.029 |
| Zinc (mg) | **10.3** | 6.1 | **10.2** | 6.0 | -0.8 | 0.006 | 0.013 |
| Copper (mg) | **1.2** | 1.4 | **1.2** | 1.4 | -1.2 | 0.006 | 0.010 |
| Iron (mg) | **14.6** | 16.7 | **14.3** | 16.6 | -2.1 | <0.001 | 0.019 |
| Potassium (mg) | **3055** | 1022 | **2993** | 986 | -2.0 | <0.001 | 0.062 |
| Sodium (mg) | **2499** | 903 | **2562** | 937 | 2.5 | <0.001 | 0.068 |
| * Calculated as the difference of the mean intake (new code – old code) divided by the mean intake using the old food codes and multiplied by 100. | | | | | | | |
|  | | | | | | | |
